# The planning, implementation and publication of a complex intervention trial for chronic fatigue syndrome: the PACE trial

**DOI:** 10.1192/pb.bp.113.045005

**Published:** 2015-02

**Authors:** Peter D. White, Trudie Chalder, Michael Sharpe

**Affiliations:** 1 Queen Mary University of London; 2 King’s College London; 3 University of Oxford

## Abstract

The PACE trial was a four-arm trial of specialist medical care, compared with specialist medical care with a supplementary therapy: adaptive pacing therapy, cognitive–behavioural therapy or graded exercise therapy, for patients with chronic fatigue syndrome. The trial found that both cognitive–behavioural and graded exercise therapies were more effective than either of the other two treatments in reducing fatigue and improving physical disability. This paper describes the design, conduct and main results of the trial, along with a description of the challenges that had to be overcome in order to produce clear answers to the clinically important questions the trial posed.

Chronic fatigue syndrome (CFS) refers to a constellation of symptoms, characterised by persistent and disabling fatigue that is typically made worse by exertion. Some believe that myalgic encephalomyelitis (ME) is another name for the same condition; others regard it as different. Whatever it is called, CFS or ME is an ‘orphan’ condition owned and cared for by no particular discipline. ME is classified within the *International Classification of Diseases* (ICD-10) as a neurological condition (G93.3),^1^ yet the majority of UK neurologists do not regard it as such.^2^ CFS may also be classified as neurasthenia (F48), a diagnosis found in the ICD-10 chapter on mental and behavioural disorders, or as a non-specific somatoform disorder.^1^ However, psychiatrists rarely see the illness as a condition that they should treat and many do not regard it as a mental illness. Therefore, while there is a consensus that CFS exists as a discrete syndrome, there is little agreement about how it should be classified, who should treat patients suffering from it, or how it should be treated.

## The PACE trial

The PACE (Pacing, graded Activity and Cognitive behaviour therapy: a randomised Evaluation) trial aimed to determine which of the non-drug treatments advocated for CFS worked best and was safe. The first research grant application to do a trial was submitted in 1998. At that time, two trials of cognitive–behavioural therapy (CBT) and two trials of graded exercise therapy (GET) had been published; these suggested that both were promising treatments, but neither treatment had been accepted by patient organisations. Therapeutic nihilism abounded and rest was often advocated as the only useful treatment.^3^

This original grant application was unsuccessful and it took a further 5 years before we obtained funding for a trial. In those 5 years, the trial design went through two more iterations. The first added a specialist medical care (SMC) arm to allow comparison of the two new therapies with the encouragement, education and support of a knowledgeable doctor. The second iteration was developed in collaboration with the patient organisation Action for ME, and added a therapy called pacing, which surveys of their members had suggested was the most helpful approach. The involvement of this patient charity, which included the involvement of the charity’s CEO as an active member of the trial management committee, was sought to ensure that the trial addressed the right questions in the right way, and to maximise the confidence of the patient community in its findings.

The concept of pacing had evolved from a strategy used in chronic pain. It is essentially an approach to management of the illness in which the patient is helped to better adapt to the symptoms by living within the limits they impose. The patient is encouraged to ‘listen to their body’ and to adopt the right balance between doing too much or too little. Pacing for CFS had never previously been tested in a trial, and the precepts behind it allowed a comparison of two different models of the illness: one adaptive (pacing), the other rehabilitative (CBT and GET). A standardised pacing therapy was produced in collaboration with Action for ME and Professor Diane Cox of Cumbria University, who agreed to be the therapy lead, having had extensive experience in delivering an occupational therapy approach to CFS that incorporated pacing. We called it ‘adaptive pacing therapy’ (APT) so as to convey the main aim of the therapy – to enable the patient to optimally adapt to the illness.

The PACE trial was finally funded in 2003.^4^

### Design issues

The PACE trial was designed to compare the efficacy and safety of SMC alone against SMC with additional APT, CBT or GET for patients with CFS recruited from six clinics in England and Scotland. We also sought to determine cost-effectiveness, mediators and moderators of outcome, as well as the patients’ long-term outcome. The trial raised a number of design issues.

#### Delivery of the therapies

A key design issue was how best to deliver the three therapies; should the same individuals deliver all three or should each be given by different people? We decided to have the most appropriate discipline deliver each therapy: CBT-trained therapists (clinical psychologists or nurse therapists) delivered CBT, physiotherapists (there was one exercise physiologist) delivered GET and occupational therapists delivered APT. This decision ensured optimal delivery of each therapy by the therapists most likely to deliver these therapies in clinical practice, but increased the risk that the relative effectiveness of the therapies could be compounded by differences in the effectiveness of the therapists themselves. Consequently, quality assurance of therapy delivery was vital. We did this in a number of ways: manuals for both therapists and patients were written and iteratively piloted, and extensive training and regular supervision of the therapists was provided.

Therapy quality was ensured by only allowing therapists to treat trial patients once their competency had been established with non-trial patients. Each therapy had a lead who delivered supervision: these were Mary Burgess, an authority on CBT for CFS; Jessica Bavinton and Lucy Clark for GET, a clinical specialist physiotherapist and a research sports physiologist, respectively; and Diane Cox for APT. All therapy sessions were audio-recorded to aid supervision and to allow ratings of therapy quality and fidelity.^5^ The treatment manuals we used in the trial are available for free download at www.pacetrial.org/trialinfo.

#### Eligibility criteria and outcomes

We decided that only patients who met the Oxford definition of CFS would be eligible to participate in the trial.^6^ This definition was widely used, broad and, unlike others, required fatigue to be the patients’ main complaint. This helped to differentiate CFS from other syndromes, such as fibromyalgia, in which fatigue is a common symptom. We were also interested in knowing whether the trial findings applied to the subgroups of patients who met alternative definitions of CFS and ME, so we stratified treatment allocation by the international criteria for CFS and also by the London criteria for ME.^7,8^

The primary outcome was hard to decide on: should it be fatigue or disability? After much debate, we chose both as co-primary outcomes because we considered them equally important aspects of the illness and potentially different in their response to the different treatments.

In order to measure treatment safety, we chose to follow the stringent European Union Clinical Trials Directive for pharmaceutical interventions, a standard rarely applied to trials of therapies.^9^ The measures of safety included adverse events, serious adverse events and reactions, withdrawal from treatments, a global self-measure of worsening, and an *a priori* threshold for deterioration in physical disability.

We also measured a number of secondary outcomes as well as potential mediators and moderators. Outcomes were assessed at baseline, mid-therapy, the end of the main treatment phase and 1-year follow-up, as well as long-term follow-up 2.5 years after randomisation.

## The analysis

Having two primary outcomes complicated the analysis, although the size of the trial gave sufficient power. Having three follow-up assessments allowed us to use a linear regression model that minimised any effects of the small amount of missing data, and also allowed us to adjust the model by factors such as baseline measures. We originally planned to use a composite outcome measure of the proportions of participants who met either a 50% reduction in the outcome score or a set threshold score for improvement. However, as we prepared our detailed statistical analysis plan, we quickly realised that a composite measure would be hard to interpret, and would not allow us to answer properly our primary questions of efficacy (i.e. comparing treatment effectiveness at reducing fatigue and disability). Before any examination of outcome data was started, and after approval by our independent steering and data monitoring committees, we decided to modify our method of analysis to one that simply compared scores between treatments at follow-up, adjusting the analysis by baseline scores. We also addressed the potential clustering effects resulting from different numbers of patients being treated by the different therapists.

## The main results

Overall, we recruited 640 patients.^10^ Almost all participants (98%) provided some outcome data, and 95% provided outcome data at 12 months, with no significant differences between arms in missing data. These very high rates of follow-up were achieved as a result of the commitment of the participants and the assiduous work by the research staff. The strategies used by the latter included offering convenient interview times (including early evenings), mailing most questionnaires to allow sufficient time to answer them before interviews, paying travel expenses, following up non-attenders expeditiously by mail and telephone, offering to see the participants at their homes and, as a last resort, recording the primary outcomes over the telephone. However, if we were doing the trial again, we would seek ethical permission to also offer follow-up by email, Skype and FaceTime.

Only 8% of participants dropped out of treatment, again with no significant difference in dropouts between treatment arms. Between 82 and 88% of participants who received a therapy alongside SMC said that they were satisfied with it, whereas only 50% reported being satisfied with SMC alone.

Analysis of the primary outcomes revealed that both CBT and GET led to significantly greater improvements in both fatigue and physical disability than did either SMC or APT. The adjusted effect sizes ranged from 0.5 to 0.8. To our surprise, there were no significant differences between APT and SMC in either primary outcome.

The differences between treatments for the secondary outcomes were broadly similar to the primary outcome comparisons. There were no significant differences in any safety measures between treatment arms. There was also a similar pattern of results in the two subgroups that met the alternative criteria for CFS and ME.

The cost of one quality-adjusted life-year (QUALY) gained was found to be £18 374 for CBT and £23 615 for GET.^11^ Both CBT and GET were three times more likely to lead to recovery from the present episode of illness than SMC alone.^12^

We concluded that both CBT and GET were moderately effective, cost-effective and safe treatments for CFS. These results were important in confirming to patients, healthcare professionals and commissioners that the promise of CBT and GET found in the earlier and small trials was justified, and that these treatments were safe to receive, if delivered as designed by the appropriate therapists.

## Implementation

The results of the trial supported the current National Institute for Health and Care Excellence (NICE) guidelines’ recommendation that ‘cognitive behavioural therapy and/or graded exercise therapy should be offered to people with mild or moderate CFS/ME [...] because currently these are the interventions for which there is the clearest research evidence of benefit.’^13^ The trial results also suggested that management by pacing, which was notably less effective than the other therapies, should not be recommended.

Now the trial has been completed and the main findings published, there is the challenging business of implementation within the National Health Service (NHS) at a time of reducing budgets. Despite the NICE guidelines suggesting that patients with long-standing CFS should be seen by specialists,^13^ many are now being sent to either Improving Access to Psychological Therapies (IAPT) services or mental health teams; these services are often neither confident nor competent in delivering CFS-specific CBT and GET. The evidence suggests that outcomes are better with specialist CFS services.^14,15^ Alternative ways of delivering therapies, such as through the internet and by telephone, which are easier to access and potentially more cost-effective, may provide ways to help patients in the future.^16,17^

## Challenges and solutions

Delivering treatment in a trial like this required the time and resources to recruit, employ, train and supervise numerous staff working across a wide geographical area. We also had to address staff turnover and the consequent extra training and supervision needed for new staff. Occasionally, it was necessary to train an existing therapist in a second therapy, which proved popular with those who did it. The morale of therapists giving a specific therapy was maintained by encouraging them to take ownership of their manualised therapy, by close supervision, and by peer support between centres.^5^

PACE was affected by several external influences. First, some patient organisations expressed opposition to the trial from the time that funding was announced.^18^ Our understanding is that this opposition reflected the fact that the trial did not focus on a biomedical approach to CFS. Meetings with those who objected did not alter their concerns and divergent views about the illness and its management continue to this day.^19^ Strategies against the trial have included a public petition to the prime minister and formal complaints to our funders and publishers.^20^ None of these complaints have been upheld, but they did take considerable time to address. A large number of Freedom of Information Act requests seeking information on all aspects of the trial have been received since the main results were published in 2011. Both declined requests that were appealed all the way up to the Information Tribunal were rejected, one being considered ‘vexatious’.^21^ There was even a debate on the trial in the House of Lords in 2013.^22^ Our deliberate policy, to help allay concerns about the trial, was to be as transparent as possible regarding what we did, while protecting medical confidentiality and our staff; this included publishing the protocol and the statistical analysis plan,^4,23^ and paying for open access to all publications. On a more positive note, some patient organisations, such as the Association for Young people with ME (AYME), have accepted the findings, and are advising their members accordingly.^24^

Second was the challenge of delivering a complex trial in the NHS. One particular threat was the ‘Agenda for Change’ which had implications for the salary grades of therapists. As a result, some of the trial centres interpreted the fact that the trial therapists had to use a manual to mean that they were unskilled, and therefore should have their pay reduced. We were successful in making the case that the trial therapists were more rather than less skilled by taking on a research role. We also supported therapists in their career progression, providing guaranteed employment beyond the trial, when possible.

The third challenge was longevity. The trial was funded in 2003; the first patient recruited in March 2005; the last patient followed up by January 2010; the main paper published in February 2011. Eight years is a long time to keep a team together and motivated. The co-principal investigators (PIs) and treatment leaders had an important role in setting the standards for trial conduct and ensuring therapy and research team cohesion and direction. In turn, the external monitoring and support of the Medical Research Council, trial steering committee and data monitoring committees were essential in maintaining the morale of the PIs. All staff met annually for a team meeting, which incorporated fun as well as training and education, supplemented by regular newsletters updating staff about progress (www.pacetrial.org/trialinfo). It may be that the external criticisms of the trial enhanced the internal cohesion and determination shown by the 100 or so staff involved.

## Conclusion

Delivering the PACE trial was an all-consuming, challenging, but ultimately rewarding task that lasted many years. We hope that it has provided useful information for patients, clinicians and commissioners about the efficacy, adverse effects and cost-effectiveness of rehabilitative interventions for CFS. We hope that we have also been able to show that it is possible to deliver a large trial of complex interventions in a challenging and sometimes hostile environment and to obtain clear results from it.
